# β-Blockers Influence Oncological Outcomes in Gastric Cancer Patients Treated with Neoadjuvant Chemotherapy Based on the Pathological Subtype: A Retrospective Cohort Study

**DOI:** 10.1245/s10434-025-17233-9

**Published:** 2025-03-25

**Authors:** Nerma Crnovrsanin, Sarah Zumsande, Ingmar Florin Rompen, Sabine Schiefer, Sarah Zimmer, Wenjun Hu, Johanna Arnscheidt, Fritz Brinkmann, Thomas Longerich, Georg Martin Haag, Thomas Schmidt, Mohammed Al-Saeedi, Leila Sisic, Henrik Nienhüser

**Affiliations:** 1 Department of General, Abdominal and Transplantation Surgery, Heidelberg University Hospital, Heidelberg, Germany; 2 Department of Pathology, Heidelberg University Hospital, Heidelberg, Germany; 3 Department of Medical Oncology, National Center of Tumor Diseases, Heidelberg University Hospital, Heidelberg, Germany; 4 Department of General, Abdominal, Tumor and Transplantation Surgery, Cologne University Hospital, Cologne, Germany; 5 Department of General, Visceral, and Transplantation Surgery, Heidelberg University Hospital, Heidelberg, Germany

**Keywords:** Gastric neoplasm, Gastric adenocarcinoma, Diffuse gastric cancer, Beta-blocker, QuPath

## Abstract

**Introduction:**

Preclinical studies suggest that β-blockers (BBs), traditionally used for cardiovascular diseases, may improve cancer outcomes. This study assessed the effect of BB intake on oncological outcomes and response to chemotherapy in gastric cancer (GC) patients and the influence of ß2-adrenergic receptor (ADRB2) expression on local tumor innervation.

**Methods:**

We retrospectively analyzed the BB intake of 361 patients who underwent surgery with curative intent for GC after neoadjuvant chemotherapy at the University Hospital of Heidelberg. Resection specimens were analyzed and immunohistochemical stainings were performed to evaluate ADRB2 expression and neuronal markers (protein gene product 9 [PGP.9]). Survival rates were estimated using Kaplan–Meier curves, and multivariable Cox regression analysis was performed to control for confounding variables.

**Results:**

In patients with diffuse GC (DGC), BB users demonstrated improved overall survival (OS) and significantly improved recurrence-free survival (RFS) compared with non-users (median OS: not reached vs. 34 months [*p* = 0.072]; median RFS: not reached vs. 16 months [*p* = 0.031]). BB intake emerged as an independent prognostic factor in multivariable analysis for this subgroup (OS: hazard ratio [HR] 0.36, 95% confidence interval [CI] 0.17–0.76; RFS: HR 0.41, 95% CI 0.20–0.87). In contrast, BB use was associated with worse OS in intestinal subtype GC (median OS: 30 months vs. not reached; *p* = 0.044), an effect that diminished after adjusting for cardiovascular risk profiles. Higher ADRB2 expression was associated with less lymph node involvement in the DGC subtype (*p* = 0.030).

**Conclusion:**

This study suggests a differential impact of BB use on GC subtypes and underscores the importance of considering cancer subtypes and patient comorbidities when evaluating the potential benefits of BBs in cancer therapy.

**Supplementary Information:**

The online version contains supplementary material available at 10.1245/s10434-025-17233-9.

Chronic stress in the form of either local or systemic inflammation can contribute to the development of cancer^1^ and has an impact on an individual’s macroenvironment through humoral and immune response changes, as well as on the tumor microenvironment through aggravation of the majority of the hallmarks of cancer, including tumor-promoting inflammation, angiogenesis, and immune resistance.^2^ In recent years, an increasing amount of literature has reported on the influence of adrenergic, cholinergic, and neurotrophin signaling pathways on these developments and has established the nervous system as an important component of the tumor microenvironment.^3–7^

Stress induced by extrinsic and intrinsic factors activates the peripheral nervous system, particularly the adrenergic pathway and ß-adrenergic receptors (ADRBs). Preclinical data indicate that adrenergic nerves send their signals through ADRBs, which activates tumor cell proliferation via the intracellular cyclic adenosine monophosphate/protein kinase A (cAMP/PKA) signaling pathway that induces further tumor-promoting pathway proteases.^8–12^ Zahalka et al. discovered a close association between the development of nerves and blood vessels in a mouse model,^4^ but adrenergic signaling can also stimulate neurotrophins and promote local tumor innervation.^11,13^ Furthermore, its activation elevates cell resistance to chemotherapeutic agents in preclinical cancer models.^14,15^

These findings suggest that β-blockers (BBs), medications used decades for cardiovascular diseases, could have an impact on response to chemotherapy and cancer-related outcomes of patients. Recent retrospective clinical studies and meta-analyses reported that patients across different types of solid tumors had a beneficial cancer outcome when taking BBs.^16–21^ Notably, a large population-based cohort study of breast cancer patients found that BBs were only associated with prolonged survival in those with triple-negative cancer subtypes.^22^ Ongoing interventional trials are investigating the addition of BBs to both conventional and new cancer therapies (NCT04005365, NCT04682158, NCT05651594).

In gastric cancer (GC), the modulation of peripheral nervous signaling by vagotomy in earlier years has highlighted the significant role of the nervous system in the development and progression of this cancer entity.^23,24^ In preclinical studies, propranolol decreased the expression of proliferation pathways and inhibited tumor growth in xenograft mouse models,^12^ while other studies highlighted the effect of β receptors on treatment efficacy. Propranolol enhanced the antitumor effects of radiotherapy in GC by inhibiting nuclear factor κB (NF-κB) expression and its downstream genes. Conversely, chronic catecholamine stimulation hindered the antitumor activities of trastuzumab in GC cells and in mice with human GC xenografts.^25,26^ While these findings appear promising, clinical studies have shown inconclusive results.^27,28^

This study aimed to evaluate the effect of BB intake on oncological outcomes and response to chemotherapy in patients with GC. Additionally, we sought to assess the impact of the ß2-adrenergic receptor (ADRB2) on tumor innervation and GC subtypes.

## Methods

Patients who underwent surgery with curative intent for gastric adenocarcinoma at the Department of General Surgery, University Hospital of Heidelberg, between April 2010 and March 2021 were included. Our study encompassed patients who met the criteria for neoadjuvant chemotherapy based on the current guidelines in Germany:^29^ patients with cT2N+ or cT3/4, and/or distant metastasis (cM1), as determined by pretherapeutic clinical staging. Patients with distant metastasis (cM1) who presented with oligometastatic disease underwent curative-intent surgery based on individual treatment decisions, as previously described.^30^ The metastatic lesions either had complete response to preoperative chemotherapy or were resected in addition to the primary tumor. All patients received preoperative chemotherapy according to either the FLOT or ECF/ECX/EOF/EOX protocols, as described previously.^31,32^ Clinicopathological and follow-up data were collected in a prospective database and analyzed retrospectively. Informed consent was obtained from all patients and the study was approved by the institutional Ethics Committee (S-635-2013).

The American Society of Anesthesiologists (ASA) physical status classification system was applied by experienced anesthesiologists and surgeons to assess medical comorbidities and perioperative risks.^33^ A total gastrectomy with D2 lymphadenectomy was performed for tumors located in the middle or distal third of the stomach. For distal GC, a subtotal gastrectomy was performed if an adequate proximal resection margin was possible. In some cases of proximal GC, a transhiatal extended gastrectomy (THG) was necessary. In patients with cM1 and intraoperative confirmation of metastasis, the surgical procedure was extended by resection of the metastatic lesions.

The histopathological work-up and response assessment was classified and staged according to the recommendation of the 8th edition of the Union for International Cancer Control (UICC) staging system; histopathological response to neoadjuvant chemotherapy was graded according to Becker et al.^34^ The resection specimens were classified into two main histopathological subtypes based on the Lauren classification—intestinal and diffuse.

The BB exposure assessment was evaluated retrospectively based on the medical records of the patients, and was counted as positive if the patient took the medication before the date of diagnosis, during their hospital stay, and after surgery.

Patients were followed according to a standardized protocol, as previously published.^35^ The last follow-up was 31 May 2022.

Overall survival (OS) was calculated from the time of diagnosis until death or last follow-up, and recurrence-free survival (RFS) was calculated from the time of surgery until disease recurrence. Survival rates were estimated using the Kaplan–Meier method, and differences in survival among the groups were calculated using the log-rank test. To compare categorical variables, we used the Chi-square and Fisher’s exact tests, and for comparison with continuous variables we used the Mann–Whitney U test or Kruskal–Wallis test, while the Pearson test was used for correlation analysis. As a post hoc analysis, a Dunn test was performed and adjusted for multiple testing using the Benjamin–Hochberg procedure. All tests were two-sided and a *p*-value <0.05 was considered statistically significant. A Cox proportional hazard regression was performed, including covariates that were significantly different between the groups, as well as known prognostic factors in GC. Analyses and figure generation were performed using R software version 4.2.2 (The R Foundation for Statistical Computing, Vienna, Austria).^36,37^

To gain further insight into the influence of ADRB2 on local tumor innervation and how it affects the different pathological subtypes of GC, we performed immunohistochemical staining for ADRB2 and protein gene product 9.5 (PGP9.5) as a marker of local innervation, and correlated them with the clinical characteristics of the patients. Detailed information on the histopathological work-up can be found in the electronic supplementary material (ESM).

## Results

### Baseline Characteristics

**Fig. 1:**
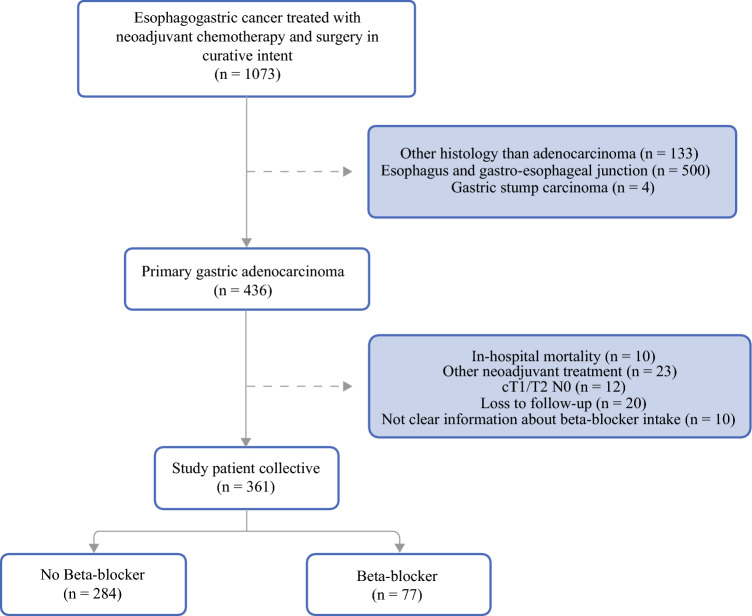
Study population

Figure 1 presents a flowchart of the patient cohort. A total of 361 GC patients were included in the final analysis, of whom 77 were BB users and 284 were BB non-users (Table 1). The usage summary of BB intake is provided in ESM Table 1. BB users were more likely to be older (68 vs. 58 years; *p* < 0.001), had a higher BMI (26.64 vs. 25.15; *p* = 0.004), more severe comorbidities (48% vs. 17%; *p* < 0.001), higher ASA classification (*p *< 0.001), more postoperative complications (*p* = 0.033), and received less adjuvant chemotherapy (64% vs. 79%; *p* = 0.006). Additionally, there was a significant difference in cT stage distribution (*p* = 0.016). Other factors such as tumor characteristics, type of neoadjuvant treatment, and complete pathological regression were not significantly different between the groups.

**Table 1:** Overview of the clinicopathological characteristics of the whole study population

Characteristic	No β-blocker [*n* = 284]	β-blocker [*n* = 77]	*p*-Value^a^
Age, years (mean [SD])	58.12 [12.87]	68.12 [9.62]	**<0.001**
*ASA classification*			**<0.001**
I/II	157 (56)	26 (34)	
III/IV	124 (44)	50 (66)	
BMI (mean [SD])	25.15 [4.41]	26.64 [4.26]	**0.004**
*Sex*			0.086
Female	123 (43)	25 (32)	
Male	161 (57)	52 (68)	
Severe comorbidities	48 (17)	37 (48)	**<0.001**
*Localization*			0.079
Cardia/fundus	56 (20)	21 (27)	
Corpus	125 (44)	23 (30)	
Antrum	89 (31)	31 (40)	
Total	14 (4.9)	2 (2.6)	
*cT*			**0.016**
1–2	12 (4.2)	6 (7.8)	
3	190 (67)	60 (78)	
4a–4b	82 (29)	11 (14)	
*cN*			0.248
cN0	40 (14)	15 (19)	
cN1–3	243 (86)	62 (81)	
*cM*			0.328
cM0	233 (82)	67 (87)	
cM1	50 (18)	10 (13)	
*Type of chemotherapy*			0.494
Epirubicin-based	88 (31)	21 (27)	
FLOT/FLO	193 (69)	56 (73)	
Interruption of chemotherapy	19 (6.8)	9 (12)	0.154
*Type of surgery*			0.603
Subtotal gastrectomy	64 (23)	20 (26)	
Total gastrectomy	149 (53)	36 (47)	
Transhiatal gastrectomy	59 (21)	16 (21)	
Extended resection	11 (3.9)	5 (6.5)	
*Clavien–Dindo*			**0.033**
0	199 (70)	42 (55)	
I/II	40 (14)	21 (27)	
IIIa/IIIb	34 (12)	11 (14)	
IVa/IVb	11 (3.9)	3 (3.9)	
*ypT*			0.823
0	14 (4.9)	4 (5.2)	
1–2	55 (19)	15 (19)	
3	130 (46)	39 (51)	
4a–4b	85 (30)	19 (25)	
*ypN*			0.746
pN0	109 (38)	28 (36)	
pN1–3	175 (62)	49 (64)	
Lymph node ratio (mean [SD])	0.18 [0.24]	0.17 [0.21]	0.990
*ypM*			0.134
pM0	239 (84)	70 (91)	
pM1	45 (16)	7 (9.1)	
*R status*			0.752
R0	224 (79)	62 (81)	
R1	60 (21)	15 (19)	
*Lauren classification*			0.160
Intestinal type	134 (47)	42 (54)	
Diffuse type	131 (46)	28 (36)	
*Pathological regression*			0.375
1a–b	69 (24)	15 (19)	
2–3	215 (76)	62 (81)	
Adjuvant treatment	219 (79)	46 (64)	**0.006**

* P* values < 0.05 were considered statistically significant and are shown in bold

Data are expressed as *n* (%) unless otherwise specified

SD, standard deviation; FLOT, fluorouracil, leucovorin, oxaliplatin, and docetaxel; FLO, fluorouracil, leucovorin, and oxaliplatin.

^a^Wilcoxon rank-sum test, Pearson’s Chi-square test, Fisher’s exact test

### Survival Analysis

The Kaplan–Meier curves for OS and RFS of the groups are shown in ESM Fig. 1. The median follow-up time for surviving patients was 49 months. The analysis did not show any significant differences between BB users and BB non-users (median OS: 57 vs. 50 months [*p* = 0.880], hazard ratio [HR] 0.96, 95% confidence interval [CI] 0.65–1.44; median RFS: not reached vs. 33 months [*p* = 0.400], HR 0.84, 95% CI 0.56–1.25). The 3-year OS was 59% in BB users compared with 57% in non-users; the 3-year RFS had similar results, with 55% in BB users and 49% in non-users. In multivariable Cox regression analysis (ESM Table 4), a positive pN status was an independent predictive factor for worse OS (HR 3.81, 95% CI 2.41–6.04; *p* < 0.001) and RFS (HR 3.09, 95% CI 2.01–4.75; *p* < 0.001). Adjuvant treatment was associated with an improved OS (HR 0.52, 95% CI 0.35–0.77; *p* = 0.001) and RFS (HR 0.62, 95% CI 0.41–0.92; *p* = 0.019).

### Subgroup Analysis

We further investigated the influence of BBs on different GC subtypes: intestinal (IGC, *n* = 176) and diffuse (DGC, *n* = 159) (ESM Tables 2 and 3). For 26 patients (7.2%), the information was not available in the final pathological report and those patients were excluded from this part of the analysis.

According to Becker et al., there were no significant differences in pathological regression between BB users and BB non-users stratified by Lauren subtype (ESM Tables 2 and 3).

**Fig. 2:**
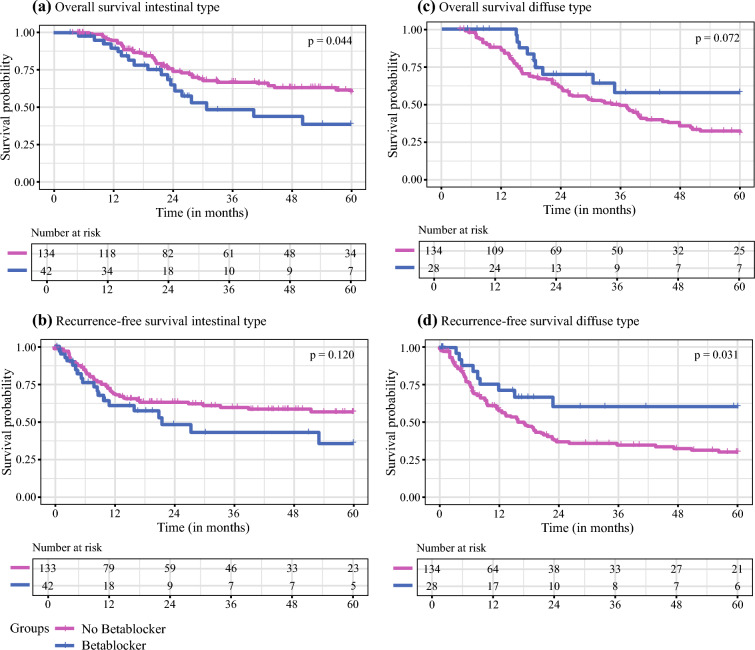
Kaplan–Meier curves and log-rank *p*-value with risk tables for **a, c** overall and **b,** **d** recurrence-free survival in patients with **a,** **b** intestinal and **c, d** diffuse gastric cancer type. A *p*-value <0.05 was considered significant

In patients with IGC, the log-rank test showed a significant result in favor of BB non-users (HR 1.70, 95% CI 1.01–3.04; *p* = 0.044) (Fig. 2a), with a median OS that was not reached and a 3-year survival of 67%; BB users had a median OS of 30 months and a 3-year survival of 49%. Contrastingly, the DGC subgroup had the opposite effect. BB users showed a trend towards improved OS (HR 0.50, 95% CI 0.27–1.07; *p* = 0.072) (Fig. 2c), with a median OS that was not reached and a 3-year survival of 58% versus a median OS of 34 months and a 3-year survival of 49% in BB non-users.

We found a similar observation in RFS (Figs. 2b, d). In the IGC group, BB users had a tendency towards worse RFS (HR 1.50, 95% CI 0.89–2.58 [*p* = 0.120]; median RFS 22 months vs. not reached, 3-year survival 43% vs. 60%), while RFS was significantly improved in the DGC group when taking BBs (HR 0.47, 95% CI 0.23–0.94 [*p* = 0.031]; median RFS not reached vs. 16 months, 3-year survival 60% vs. 35%).

We conducted a multivariable Cox regression analysis across the subgroups and observed similar results (Tables 2 and 3). BB use was an independent predictive factor for improved OS (HR 0.36, 95% CI 0.17–0.76; *p* = 0.008) and RFS (HR 0.41, 95% CI 0.20–0.87; *p* = 0.019) in patients with DGC.

**Table 2:**
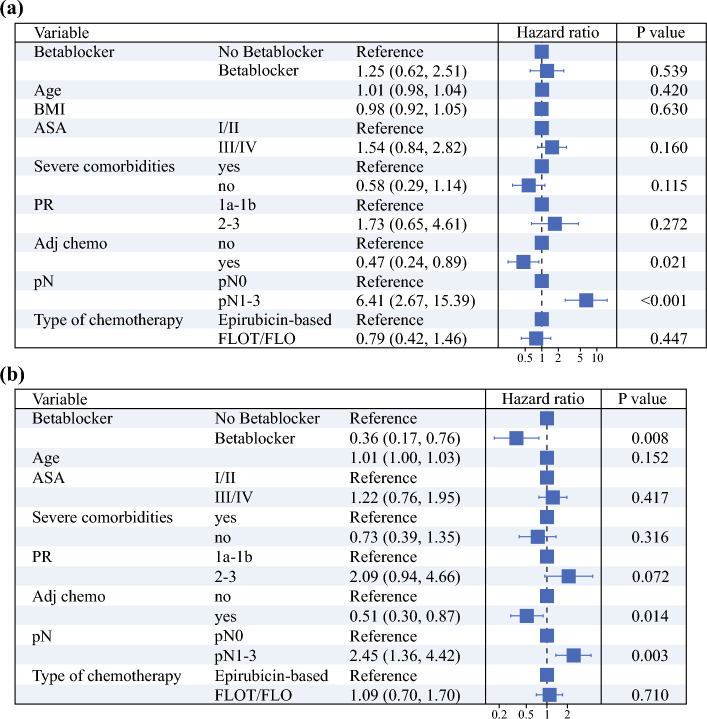
Forest plot of the multivariable Cox regression for overall survival

Results of the multivariable Cox regression in the subgroup of patients with (**a**) intestinal type and (**b**) diffuse type

BMI, body mass index; ASA, American Society of Anesthesiologists; PR, pathological response; Adj, chemo adjuvant chemotherapy received; FLOT, fluorouracil, leucovorin, oxaliplatin, and docetaxel; FLO, fluorouracil, leucovorin, and oxaliplatin

**Table 3:**
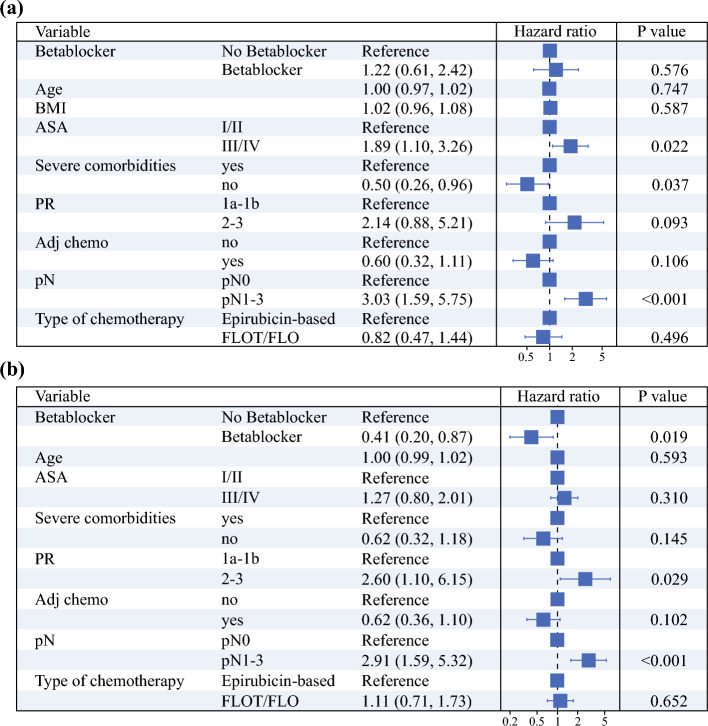
Forest plot of the Cox regression for recurrence-free survival

Results of the multivariable Cox regression in the subgroup of patients with (**a**) intestinal type and (**b**) diffuse type

BMI, body mass index; ASA, American Society of Anesthesiologists; PR, pathological response; Adj, chemo adjuvant chemotherapy received; FLOT, fluorouracil, leucovorin, oxaliplatin, and docetaxel; FLO, fluorouracil, leucovorin, and oxaliplatin

### Immunohistochemical Analysis

Patient characteristics for those with available primary tumor tissue (*n* = 142) are reported in ESM Table 5, and descriptive statistics of the analyzed markers are reported in Table 4.

**Table 4:** Descriptive statistics of the markers ADRB2 and PGP

Characteristic	β-blocker, [*n* = 21]	No β-blocker [*n* = 121]	*p*-Value^a^	Overall [*n* = 142]
*ADRB expression*			**0.011**	
Median (SD)	0.179 (0.113)	0.065 (0.102)		0.078 (0.105)
Range	0.006, 0.466	0.002, 0.515		0.002, 0.515
*PGP expression*			0.700	
Median (SD)	0.002 (0.006)	0.001 (0.025)		0.002 (0.023)
Range	0.000, 0.023	0.000, 0.245		0.000, 0.245

*P* values < 0.05 are considered significant and are shown in bold

^a^Wilcoxon rank-sum test

SD, standard deviation.

We observed that BB users had a significantly higher expression of ADRB2 than non-users (median 0.179 vs. 0.065; *p* = 0.011), suggesting an upregulation of the receptor under therapy. We did not observe any association with the local innervation (*p* = 0.700).

**Fig. 3:**
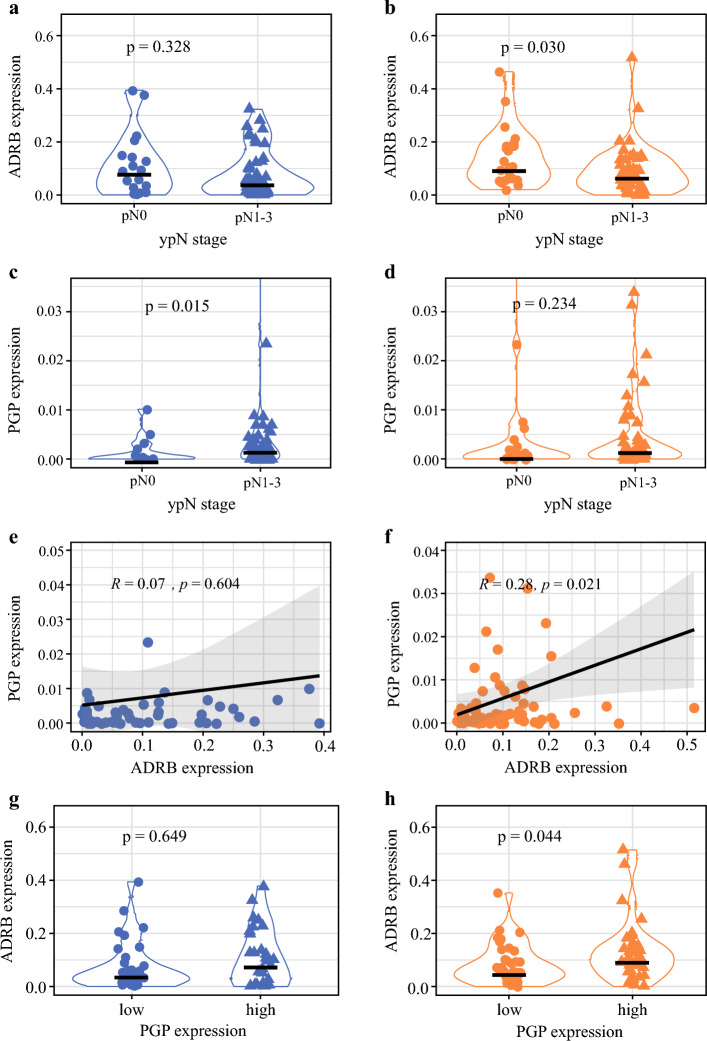
Violin plots illustrating the association between ADRB2 expression, PGP expression, and pN stage in patients with **a,** **c,** **e,** **g** IGC and **b,** **d,** **f,** **h** DGC. a, b Hypothesis test comparing pN stage with ADRB expression. c, d Hypothesis test comparing pN stage with PGP expression. e, f Pearson correlation between ADRB and PGP expression. g, h Hypothesis test comparing low and high PGP expression (grouped according to the median) with ADRB expression. A *p*-value <0.05 is considered significant. *ADRB2* ß2-adrenergic receptor, *IGC* intestinal type gastric cancer, *DGC* diffuse type gastric cancer, *ADRB* ß-adrenergic receptor, *PGP* protein gene product

We further examined ADRB2 expression in the DGC subgroup (*n* = 67) and observed that a high ADRB2 expression was associated with ypN0 stage (*p* = 0.030) (Fig. 3b); however, there was no association in the IGC subgroup (*p* = 0.328) (Fig. 3a). Additionally, ADRB2 expression showed a significant correlation with PGP9.5 expression in DGC (*r* = 0.28, *p* = 0.021) (Fig. 3f), but this was not observed in the IGC subgroup (*r* = 0.07, *p* = 0.604) (Fig. 3e). This observation was similar if PGP9.5 expression was treated as a categorical variable, divided by the median in high and low PGP9.5 expression (Fig. 3g and h).

On the other hand, high PGP9.5 expression was significantly associated with advanced ypN stage in IGC patients (Fig. 3c). The median PGP9.5 expression was 0.03‰ in ypN0 (*n* = 18) versus 0.22‰ in ypN1-3 (*n* = 44) in patients with IGC (*p* = 0.015) (ESM Table 6c, Fig. 4c). However, this effect was attenuated in patients with DGC; the median PGP9.5 was 0.09‰ in pN0 (*n* = 21) versus 0.21‰ in pN1-3 (*n* = 46; *p* = 0.234) (ESM Table 6d, Fig. 4d).

Due to the limited number of patients in the BB group, we could not differentiate between BB users (*n* = 21: IGC, *n* = 15; DGC, *n* = 3) and BB non-users (*n* = 121: IGC, *n* = 48; DGC, *n* = 62) (ESM Table 5). As BB use is a confounding factor in ADRB2 expression, we performed an analysis on the subgroup of patients who did not take BBs and observed significantly higher ADRB2 expression in the DGC subgroup compared with the IGC subgroup (data not shown).

## Discussion

This clinicopathological study represents the first and largest evaluation of the prognostic significance of BB treatment and β-receptor expression in European GC patients. The primary objective was to assess whether the use of BBs was associated with oncological outcomes and response to chemotherapy in patients with GC treated with neoadjuvant chemotherapy. Although there were no differences in outcomes in the overall study population, patients in the DGC subgroup taking BBs exhibited improved OS and RFS. Additionally, BB use was identified as an independent prognostic factor for enhanced OS and RFS in this subgroup but did not have any impact on the response to chemotherapy. Moreover, immunohistochemical staining revealed that higher ADRB2 expression was associated with BB use in all patients, while in the DGC subgroup, higher ADRB2 expression was linked to a lower pN stage but increased local tumor innervation.

Although the effects of β-adrenergic signaling and its inhibition on gastric tumor progression and metastasis seems to be well established in preclinical models,^12,23,38,39^ little clinical data exist to confirm these observations in comparison with other types of tumors.^17,18,40^ Shah et al. analyzed 93 patients with GC and found no significant differences in OS between BB users and non-users (HR 1.44, 95% CI 0.76–2.74). Additionally, no significant differences were noted between the use of selective and non-selective BBs.^41^ Our study corroborated these findings, showing no benefits in OS and RFS among the entire study population. Furthermore, there was no significant impact of BB use on histopathological tumor response. However, it is important to note that BB users were significantly older and had a higher ASA classification than non-users.

In contrast to the non-significant findings in the overall population, we observed significant differences in survival when observing the subgroups based on histopathological subtypes. In DGC patients, BB intake was linked to improved OS and RFS, and emerged as an independent prognostic factor in the multivariable analysis after adjusting for differences between the groups. However, in the IGC subgroup, we observed the opposite effects, with BB use associated with worse OS. This effect diminished when we adjusted for confounding factors, primarily driven by differences in comorbidities.

The expression of β-adrenergic receptors in human GC tissues and their correlation with clinical outcomes is examined in a substantial number of studies.^12,38,42,43^ Koh et al. showed that in 162 patients with stage I–III GC, high ADRB2 expression was significantly associated with pT, pN, venous invasion, and tumor stage. There were also significant differences in both OS and RFS between patients with high and low ADRB2 expression, and the multivariable analysis confirmed that pT, pN, and ADRB2 expression were independent prognostic factors of poor RFS.^12^ In the study by Takahashi et al., higher ADRB2 expression was associated with advanced age, tumor aggressiveness, and histology of non-signet ring cells.^42^ Our study also revealed differences in ADRB2 expression based on histopathological subtypes. In patients with DGC subtypes, higher ADRB2 expression was associated with ypN0 stage and increased local innervation measured by the PGP9.5 marker. In IGC, there was no such association with ADRB2 expression, but a higher local innervation was associated with a positive pN stage. This suggests a more significant role of ADRB2 receptors in lymphatic metastasis and local innervation in DGC.

DGC subtype with or without signet ring cells exhibits the characteristics of high malignancy, poor response to chemotherapy, higher risk of metastasis, and much younger age at diagnosis.^44^ The use of BBs in this subgroup of GC patients is poorly supported by the current literature. In the above-mentioned study by Takahashi et al., 41 patients had signet ring cells and just one had high ADRB2 expression.^42^ Koh et al. included 87 patients with signet ring cells and the majority showed high ADRB2 expression; however, they did not perform separate survival analysis based on the subtypes.

In our study, the notable differences in the effect of BBs on these two subgroups might be due to different reasons. First, cancer patients with more comorbidities have poorer oncological outcomes than those with or without less severe comorbidities.^45^ In our study, BB users in the IGC subgroup were older and had more severe comorbidities than BB users in the DGC subgroup.

Second, there are differences in the tumor biology and pathogenesis of these two subgroups, and some literature suggests treating them as two distinct types of tumors in general.^46^ A recently published work using single-cell sequencing of DGC revealed insight into the biology of this tumor entity. Based on the gene set enrichment analysis (GSEA), upregulated genes were mainly enriched in the immune response, such as tumor necrosis factor (TNF)-α signaling via the NF-kB signaling pathway, among others. Additionally, cell proliferation-related signaling pathways such as mitogen-activated protein kinase (MAPK) were also enriched.^44^ cAMP is the best-characterized signaling pathway that regulates the activation of MAPKs. This cAMP activation is induced through PKA signaling, which also represents the main pathway of β-adrenergic receptors.^11,47^ The BBs might also modulate the immune response and inflammation induced by NF-kB.^10,48^ On the other hand, intestinal gastric adenocarcinoma has shown, in both single cell as well as bulk RNA-seq studies, lower immune scores.^49,50^ However, more research is currently needed to further explore these differences, and our study might contribute to more evidence in this field and the generation of hypotheses.

Despite these novel findings, this study has several limitations. The major limitation is that almost all patients took selective BBs such as metoprolol or bisoprolol. Only a very small number of patients received non-selective BBs, making a separate analysis insufficiently powered for a valid conclusion. However, with increasing dose, selective BBs have an increasingly unselective effect, as seen in similar studies.^28,51^ Second, the retrospective collection of information regarding BB intake raises uncertainty about patients taking BBs regularly. There might also be confounding factors related to other pharmacological exposures that could affect cancer progression.^52–54^ Additionally, the exact percentage of signet ring cells in the DGC subtypes was not reported in the final pathological reports. Lastly, it would have been interesting to correlate the results with perineural invasion of the surgical specimens and molecular subtypes, particularly the microsatellite instability subtypes or combined positive scores. Unfortunately, this information was not available, however we did perform staining for PGP9.5 to assess innervation, which showed no association with BB use.

Nevertheless, our study represents the largest assessment of BB intake in an homogenous, Caucasian GC patient cohort, providing valuable evidence in the emerging field of cancer neurogenesis.

## Conclusion

Within the context of GC, this study offers new insight into neurogenesis and contributes to a larger body of evidence suggesting that the β-adrenergic pathway has a more important role in diffuse GC. By highlighting the potential influence of the nervous system on cancer progression, our findings provide a valuable foundation for further preclinical and ongoing clinical trials in this field.

## Supplementary Information

Below is the link to the electronic supplementary material.

Supplementary file1 (DOCX 463 KB)

Supplementary file2 (TIF 4690 KB)

## Data Availability

The datasets generated and/or analyzed during the current study are available from the corresponding author upon reasonable request. Due to privacy and ethical considerations, some restrictions may apply to the availability of these data.
