# Supplementary material for: β-Blockers Influence Oncological Outcomes in Gastric Cancer Patients Treated with Neoadjuvant Chemotherapy Based on the Pathological Subtype: A Retrospective Cohort Study
Source: Ann Surg Oncol. 2025 Mar 25;32(7):5142–53. doi: 10.1245/s10434-025-17233-9 (PMC12129865; doi:10.1245/s10434-025-17233-9)
Supplement: Supplementary file 1 — Supplementary file1 (DOCX 463 KB) [file 10434_2025_17233_MOESM1_ESM.docx]

**Supplementary**

**Supp.Fig.1** Kaplan-Meier curve of the a) overall and b) recurrence-free survival comparing BB-users and BB-non-users of the whole study population.

**Supp.Table 1** Beta-blocker characteristics and Usage Summary.

| Characteristic | n = 77 |
| --- | --- |
| Reason of intake |  |
| Coronary heart disease | 12 (16%) |
| Heart failure | 1 (1.3%) |
| Tachycardic arrhythmias | 5 (6.5%) |
| Hypertension | 44 (57%) |
| Multiple reasons | 15 (19%) |
| Specific name of the medication |  |
| Atenolol | 1 (1.3%) |
| Bisoprolol | 31 (40%) |
| Carvedilol | 3 (3.9%) |
| Metoprolol | 38 (49%) |
| Nebivolol | 3 (3.9%) |
| Sotalol | 1 (1.3%) |
| Selectivity |  |
| nonselective | 3 (3.9%) |
| selective | 74 (96%) |
| Dosage of the medication |  |
| high | 29 (38%) |
| low | 48 (62%) |
| n (%) | |

**Supp. Table 2** Overview of the clinicopathological characteristics of the subgroup with intestinal GC.

| **Characteristic** | **No Betablocker**,  n = 134^1^ | **Betablocker**,  n = 42^1^ | **p-value**^2^ |
| --- | --- | --- | --- |
| **Age** | 61.60 (11.33) | 70.40 (8.66) | **<0.001** |
| **ASA classification** |  |  | **0.016** |
| I/II | 73 / (56%) | 14 / (34%) |  |
| III/IV | 58 / (44%) | 27 / (66%) |  |
| **BMI** | 25.17 (4.18) | 26.90 (4.03) | **0.016** |
| **Sex** |  |  | 0.202 |
| female | 46 / (34%) | 10 / (24%) |  |
| male | 88 / (66%) | 32 / (76%) |  |
| **Severe comorbidities** | 29 / (22%) | 22 / (52%) | **<0.001** |
| **Localization** |  |  | 0.169 |
| Cardia/Fundus | 31 / (23%) | 13 / (31%) |  |
| Corpus | 61 / (46%) | 12 / (29%) |  |
| Antrum | 41 / (31%) | 16 / (38%) |  |
| Total | 1 / (0.7%) | 1 / (2.4%) |  |
| **cT** |  |  | 0.100 |
| 1-2 | 8 / (6.0%) | 5 / (12%) |  |
| 3 | 84 / (63%) | 30 / (71%) |  |
| 4a-4b | 42 / (31%) | 7 / (17%) |  |
| **cN** |  |  | 0.999 |
| cN0 | 15 / (11%) | 4 / (9.5%) |  |
| cN1-3 | 118 / (89%) | 38 / (90%) |  |
| **cM** |  |  | 0.310 |
| cM0 | 109 / (81%) | 37 / (88%) |  |
| cM1 | 25 / (19%) | 5 / (12%) |  |
| **Type of chemo** |  |  | 0.627 |
| Epirubicin - based | 27 / (20%) | 10 / (24%) |  |
| FLOT/FLO | 106 / (80%) | 32 / (76%) |  |
| **Interruption of chemo** | 8 / (6.1%) | 7 / (17%) | 0.053 |
| **Type of surgery** |  |  | 0.268 |
| Subtotal Gastrectomy | 30 / (23%) | 10 / (24%) |  |
| Total gastrectomy | 70 / (53%) | 17 / (40%) |  |
| Transhiatal gastrectomy | 27 / (20%) | 10 / (24%) |  |
| Extended resection | 6 / (4.5%) | 5 / (12%) |  |
| **Clavien-Dindo** |  |  | 0.208 |
| 0 | 100 / (75%) | 25 / (60%) |  |
| I/II | 21 / (16%) | 10 / (24%) |  |
| IIIa/IIIb | 10 / (7.5%) | 6 / (14%) |  |
| IVa/IVb | 3 / (2.2%) | 1 / (2.4%) |  |
| **pT** |  |  | 0.649 |
| 0 | 7 / (5.2%) | 2 / (4.8%) |  |
| 1-2 | 31 / (23%) | 7 / (17%) |  |
| 3 | 58 / (43%) | 23 / (55%) |  |
| 4a-4b | 38 / (28%) | 10 / (24%) |  |
| **ypN** |  |  | 0.181 |
| pN0 | 57 / (43%) | 13 / (31%) |  |
| pN1-3 | 77 / (57%) | 29 / (69%) |  |
| **Lymph node ratio** | 0.15 (0.21) | 0.18 (0.18) | 0.084 |
| **ypM** |  |  | 0.766 |
| pM0 | 120 / (90%) | 39 / (93%) |  |
| pM1 | 14 / (10%) | 3 / (7.1%) |  |
| **R status** |  |  | 0.995 |
| 0 | 118 / (88%) | 37 / (88%) |  |
| 1 | 16 / (12%) | 5 / (12%) |  |
| **Pathological Regression** |  |  | 0.079 |
| 1a-1b | 37 / (28%) | 6 / (14%) |  |
| 2-3 | 97 / (72%) | 36 / (86%) |  |
| **Adjuvant treatment** | 99 / (77%) | 23 / (62%) | 0.064 |
| ^1^Mean (SD); n / (%) | | | |
| ^2^Wilcoxon rank sum test; Pearson's Chi-squared test; Fisher's exact test | | | |

**Supp. Table 3** Overview of the clinicopathological characteristics of the subgroup with diffuse GC.

| **Characteristic** | **No Betablocker**,  n = 131^1^ | **Betablocker**,  n = 28^1^ | **p-value**^2^ |
| --- | --- | --- | --- |
| **Age** | 54.86 (13.56) | 63.82 (10.04) | **0.001** |
| **ASA classification** |  |  | **0.046** |
| I/II | 74 / (56%) | 10 / (36%) |  |
| III/IV | 57 / (44%) | 18 / (64%) |  |
| **BMI** | 25.28 (4.83) | 26.63 (4.77) | 0.137 |
| **Sex** |  |  | 0.309 |
| female | 70 / (53%) | 12 / (43%) |  |
| male | 61 / (47%) | 16 / (57%) |  |
| **Severe comorbidities** | 17 / (13%) | 9 / (32%) | **0.023** |
| **Localization** |  |  | 0.530 |
| Cardia/Fundus | 19 / (15%) | 7 / (25%) |  |
| Corpus | 60 / (46%) | 11 / (39%) |  |
| Antrum | 41 / (31%) | 9 / (32%) |  |
| Total | 11 / (8.4%) | 1 / (3.6%) |  |
| **cT** |  |  | 0.304 |
| 1-2 | 4 / (3.1%) | 0 / (0%) |  |
| 3 | 92 / (70%) | 24 / (86%) |  |
| 4a-4b | 35 / (27%) | 4 / (14%) |  |
| **cN** |  |  | 0.063 |
| cN0 | 22 / (17%) | 9 / (32%) |  |
| cN1-3 | 109 / (83%) | 19 / (68%) |  |
| **cM** |  |  | >0.999 |
| cM0 | 108 / (83%) | 23 / (82%) |  |
| cM1 | 22 / (17%) | 5 / (18%) |  |
| **Type of chemo** |  |  | 0.247 |
| Epirubicin - based | 52 / (40%) | 8 / (29%) |  |
| FLOT/FLO | 77 / (60%) | 20 / (71%) |  |
| **Interruption of chemo** | 10 / (7.7%) | 2 / (7.1%) | >0.999 |
| **Type of surgery** |  |  | 0.843 |
| Subtotal Gastrectomy | 29 / (22%) | 5 / (18%) |  |
| Total gastrectomy | 73 / (56%) | 18 / (64%) |  |
| Transhiatal gastrectomy | 24 / (18%) | 5 / (18%) |  |
| Extended resection | 5 / (3.8%) | 0 / (0%) |  |
| **Clavien-Dindo** |  |  | 0.294 |
| 0 | 88 / (67%) | 15 / (54%) |  |
| I/II | 13 / (9.9%) | 6 / (21%) |  |
| IIIa/IIIb | 22 / (17%) | 5 / (18%) |  |
| IVa/IVb | 8 / (6.1%) | 2 / (7.1%) |  |
| **pT** |  |  | 0.443 |
| 1-2 | 20 / (15%) | 7 / (25%) |  |
| 3 | 68 / (52%) | 12 / (43%) |  |
| 4a-4b | 43 / (33%) | 9 / (32%) |  |
| **ypN** |  |  | 0.325 |
| pN0 | 39 / (30%) | 11 / (39%) |  |
| pN1-3 | 92 / (70%) | 17 / (61%) |  |
| **Lymph node ratio** | 0.23 (0.26) | 0.16 (0.26) | 0.099 |
| **ypM** |  |  | 0.396 |
| pM0 | 103 / (79%) | 24 / (86%) |  |
| pM1 | 28 / (21%) | 4 / (14%) |  |
| **R status** |  |  | 0.768 |
| 0 | 88 / (67%) | 18 / (64%) |  |
| 1 | 43 / (33%) | 10 / (36%) |  |
| **Pathological Regression** |  |  | >0.999 |
| 1a-1b | 22 / (17%) | 5 / (18%) |  |
| 2-3 | 109 / (83%) | 23 / (82%) |  |
| **Adjuvant treatment** | 105 / (81%) | 18 / (64%) | **0.046** |
| ^1^Mean (SD); n / (%) | | | |
| ^2^Wilcoxon rank sum test; Pearson's Chi-squared test; Fisher's exact test | | | |

**Supp. Table 4** Results of the multivariable cox regression of the whole study population for a) overall survival and b) recurrence-free survival. PR = Pathological response, Adj chemo = adjuvant chemotherapy received

a)


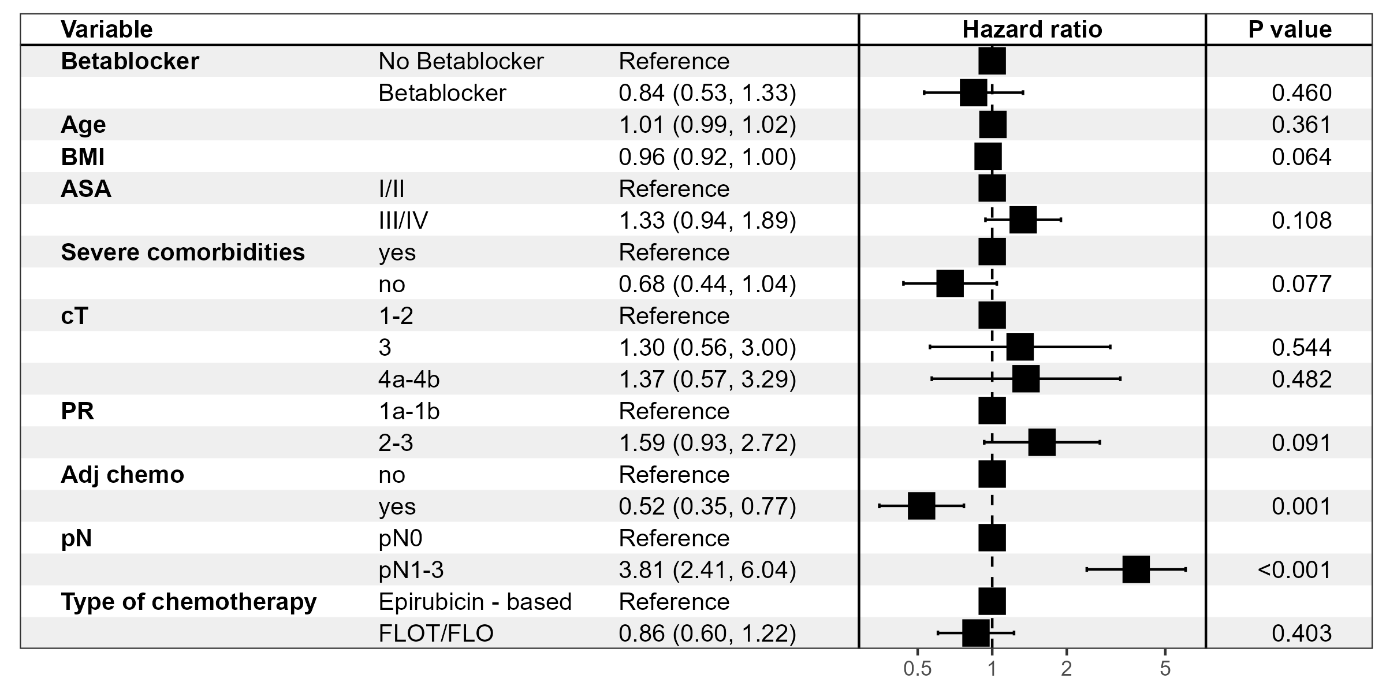


b)


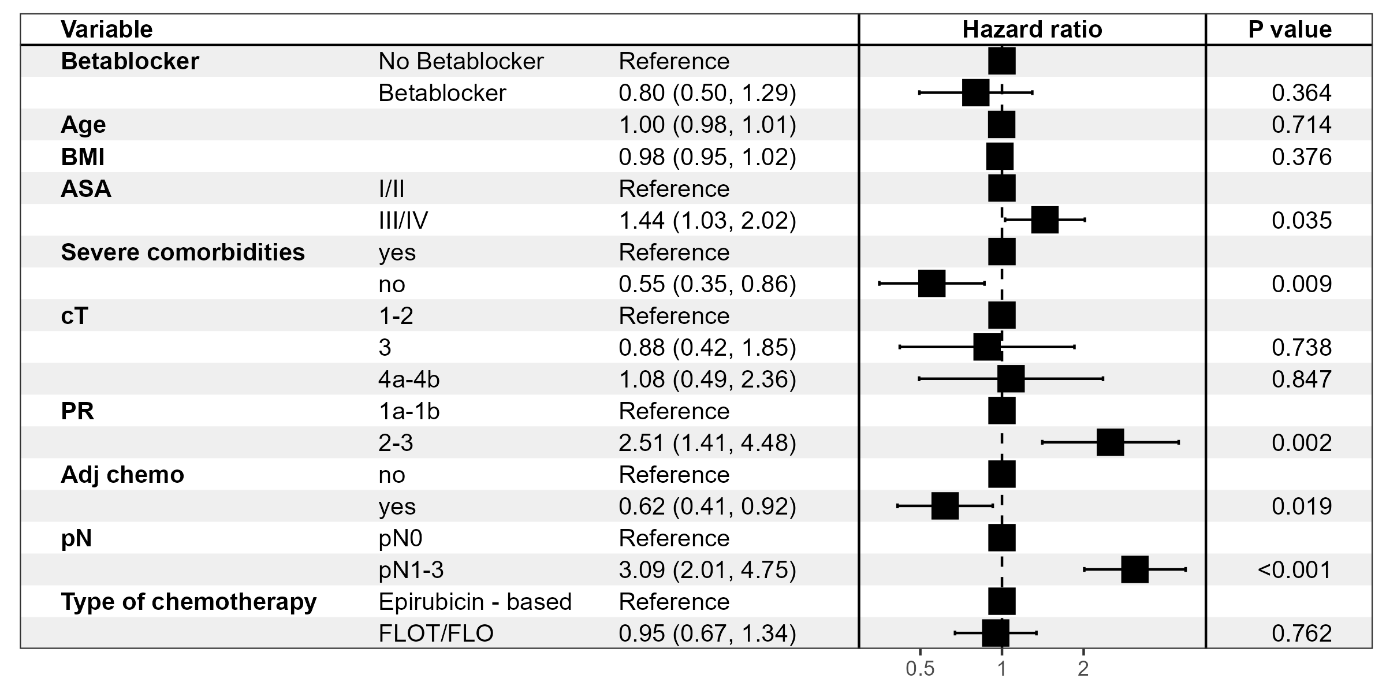


**Supp. Table 5** Overview of the clinicopathological characteristics of patients where pathological slides were available.

| **Characteristic** | **N = 142**^1^ |
| --- | --- |
| **Age** | 59.60 (13.89) |
| **ASA classification** |  |
| I/II | 85 (60%) |
| III/IV | 56 (40%) |
| **BMI** | 25.50 (4.19) |
| **Sex** |  |
| female | 57 (40%) |
| male | 85 (60%) |
| **Betablocker** |  |
| Betablocker | 21 (15%) |
| No Betablocker | 121 (85%) |
| **Severe comorbidities** | 23 (16%) |
| **Localization** |  |
| Antrum | 42 (30%) |
| Cardia/Fundus | 33 (23%) |
| Corpus | 61 (43%) |
| Total | 6 (4.2%) |
| **cN** |  |
| cN0 | 23 (16%) |
| cN1-3 | 119 (84%) |
| **cM** |  |
| cM0 | 123 (87%) |
| cM1 | 19 (13%) |
| **Type of chemo** |  |
| Epirubicin - based | 42 (30%) |
| FLOT/FLO | 99 (70%) |
| **Type of surgery** |  |
| Extended resection | 11 (7.8%) |
| Subtotal Gastrectomy | 30 (21%) |
| Total gastrectomy | 65 (46%) |
| Transhiatal gastrectomy | 35 (25%) |
| **Clavien-Dindo** |  |
| 0 | 87 (61%) |
| I/II | 24 (17%) |
| IIIa/IIIb | 23 (16%) |
| IVa/IVb | 8 (5.6%) |
| **ypT** |  |
| 0 | 6 (4.2%) |
| 1-2 | 25 (18%) |
| 3 | 72 (51%) |
| 4a-4b | 39 (27%) |
| **ypN** |  |
| pN0 | 48 (34%) |
| pN1-3 | 94 (66%) |
| **Lymph node ratio** | 0.20 (0.22) |
| **ypM** |  |
| pM0 | 122 (86%) |
| pM1 | 20 (14%) |
| **R status** |  |
| R0 | 112 (79%) |
| R1 | 30 (21%) |
| **Lauren classification** |  |
| Intestinal type | 62 (43%) |
| Diffuse type | 67 (47%) |
| **Pathological Regression** |  |
| 1a-1b | 31 (22%) |
| 2-3 | 111 (78%) |
| **Adjuvant treatment** | 109 (78%) |
| ^1^Mean (SD); n (%) | |

**Supp.Table 6**

a) Association of ADRB2 expression with clinical and pathological characteristics in patients with intestinal GC.

| Variable | comparison | Median expression ADRB | Median expression ADRB | R | p-value |
| --- | --- | --- | --- | --- | --- |
| BMI |  |  |  | 0.264 | **0.038** |
| Age |  |  |  | -0.054 | 0.674 |
| Lymph node ratio | |  |  | -0.013 | 0.917 |
| Betablocker | Betablocker - No Betablocker | 0.13 | 0.05 |  | 0.058 |
| ASA | I/II - III/IV | 0.06 | 0.03 |  | 0.433 |
| Sex | female - male | 0.04 | 0.06 |  | 0.284 |
| Localization | Antrum - Cardia/Fundus | 0.05 | 0.07 |  | 0.613 |
|  | Antrum - Corpus | 0.05 | 0.06 |  | 0.861 |
|  | Cardia/Fundus - Corpus | 0.07 | 0.06 |  | 0.553 |
|  | Antrum - Total | 0.05 | 0.01 |  | 0.736 |
|  | Cardia/Fundus - Total | 0.07 | 0.01 |  | 1.000 |
|  | Corpus - Total | 0.06 | 0.01 |  | 1.000 |
| Type of chemo | Epirubicin - based - FLOT/FLO | 0.05 | 0.06 |  | 0.893 |
| ypT | 0 - 1/2 | 0.06 | 0.04 |  | 1.000 |
|  | 0 – 3 | 0.06 | 0.06 |  | 0.942 |
|  | 1/2 – 3 | 0.04 | 0.06 |  | 1.000 |
|  | 0 - 4a/b | 0.06 | 0.05 |  | 1.000 |
|  | 1/2 - 4a/b | 0.04 | 0.05 |  | 0.983 |
|  | 3 - 4a/b | 0.06 | 0.05 |  | 1.000 |
| ypN | pN1-3 - pN0 | 0.05 | 0.09 |  | 0.328 |
| ypM | pM1 - pM0 | 0.06 | 0.06 |  | 0.625 |
| R status | R0 - R1 | 0.06 | 0.08 |  | 0.562 |
| Pathological regression | 2/3 - 1a/1b | 0.06 | 0.05 |  | 0.822 |

b) Association of ADRB2 expression with clinical and pathological characteristics in patients with diffuse GC.

| Variable | comparison | Median expression ADRB | Median expression ADRB | R | p-value |
| --- | --- | --- | --- | --- | --- |
| BMI |  |  |  | 0.139 | 0.267 |
| Age |  |  |  | -0.129 | 0.300 |
| Lymph node ratio | |  |  | -0.187 | 0.130 |
| Betablocker | Betablocker - No Betablocker | 0.15 | 0.09 |  | 0.268 |
| ASA | I/II - III/IV | 0.09 | 0.09 |  | 0.638 |
| Sex | female - male | 0.09 | 0.07 |  | 0.570 |
| Localization | Antrum - Cardia/Fundus | 0.06 | 0.07 |  | 0.828 |
|  | Antrum - Corpus | 0.06 | 0.09 |  | 1.000 |
|  | Cardia/Fundus - Corpus | 0.07 | 0.09 |  | 1.000 |
|  | Antrum - Total | 0.06 | 0.09 |  | 1.000 |
|  | Cardia/Fundus - Total | 0.07 | 0.09 |  | 0.867 |
|  | Corpus - Total | 0.09 | 0.09 |  | 0.950 |
| Type of chemo | Epirubicin - based - FLOT/FLO | 0.08 | 0.09 |  | 0.146 |
| ypT | 1/2 – 3 | 0.12 | 0.08 |  | 0.781 |
|  | 1/2 - 4a/b | 0.12 | 0.09 |  | 0.692 |
|  | 3 - 4a/b | 0.08 | 0.09 |  | 0.676 |
| ypN | pN1-3 - pN0 | 0.08 | 0.11 |  | **0.030** |
| ypM | pM1 - pM0 | 0.13 | 0.08 |  | 0.130 |
| R status | R0 - R1 | 0.09 | 0.09 |  | 0.732 |
| Pathological Regression | 2/3 - 1a/1b | 0.08 | 0.13 |  | 0.195 |

c) Association of PGP expression with clinical and pathological characteristics in patients with intestinal GC.

| Variable | comparison | Median expression PGP | Median expression PGP | R | p-value |
| --- | --- | --- | --- | --- | --- |
| BMI |  |  |  | 0.143 | 0.268 |
| Age |  |  |  | -0.057 | 0.660 |
| Lymph node ratio | |  |  | 0.196 | 0.126 |
| Betablocker | Betablocker - No Betablocker | 0.0019 | 0.0008 |  | 0.565 |
| ASA | I/II - III/IV | 0.0016 | 0.0012 |  | 0.796 |
| Sex | female - male | 0.0021 | 0.0008 |  | 0.433 |
| Localization | Antrum - Cardia/Fundus | 0.0019 | 0.0011 |  | 0.695 |
|  | Antrum - Corpus | 0.0019 | 0.0008 |  | 0.695 |
|  | Cardia/Fundus - Corpus | 0.0011 | 0.0008 |  | 0.826 |
|  | Antrum - Total | 0.0019 | 0.0001 |  | 1.000 |
|  | Cardia/Fundus - Total | 0.0011 | 0.0001 |  | 0.730 |
|  | Corpus - Total | 0.0008 | 0.0001 |  | 0.911 |
| Type of chemo | Epirubicin - based - FLOT/FLO | 0.0034 | 0.0008 |  | 0.074 |
| ypT | 0 - 1/2 | 0.0002 | 0.0003 |  | 0.768 |
|  | 0 – 3 | 0.0002 | 0.0021 |  | 0.821 |
|  | 1/2 – 3 | 0.0003 | 0.0021 |  | 1.000 |
|  | 0 - 4a/b | 0.0002 | 0.0006 |  | 0.563 |
|  | 1/2 - 4a/b | 0.0003 | 0.0006 |  | 0.734 |
|  | 3 - 4a/b | 0.0021 | 0.0006 |  | 0.884 |
| ypN | pN1-3 - pN0 | 0.0022 | 0.0003 |  | **0.015** |
| ypM | pM1 - pM0 | 0.0005 | 0.0016 |  | 0.266 |
| R status | R0 - R1 | 0.0019 | 0.0006 |  | 0.617 |
| Pathological Regression | 2/3 - 1a/1b | 0.0016 | 0.0005 |  | 0.216 |

d) Association of PGP expression with clinical and pathological characteristics in patients with diffuse GC.

| Variable | comparison | Median expression PGP | Median expression PGP | R | p-value |
| --- | --- | --- | --- | --- | --- |
| BMI |  |  |  | -0.008 | 0.947 |
| Age |  |  |  | 0.160 | 0.196 |
| Lymph node ratio | |  |  | 0.153 | 0.217 |
| Betablocker | Betablocker - No Betablocker | 0.0008 | 0.0016 |  | 0.431 |
| ASA | I/II - III/IV | 0.0014 | 0.0022 |  | 0.979 |
| Sex | female - male | 0.0021 | 0.0014 |  | 0.339 |
| Localization | Antrum - Cardia/Fundus | 0.0006 | 0.0029 |  | 0.337 |
|  | Antrum - Corpus | 0.0006 | 0.0022 |  | 0.361 |
|  | Cardia/Fundus - Corpus | 0.0029 | 0.0022 |  | 0.943 |
|  | Antrum - Total | 0.0006 | 0.0022 |  | 0.471 |
|  | Cardia/Fundus - Total | 0.0029 | 0.0022 |  | 0.751 |
|  | Corpus - Total | 0.0022 | 0.0022 |  | 0.838 |
| Type of chemo | Epirubicin - based - FLOT/FLO | 0.0012 | 0.0019 |  | 0.610 |
| ypT | 1/2 - 3 | 0.0008 | 0.0014 |  | 0.598 |
|  | 1/2 - 4a/b | 0.0008 | 0.0022 |  | 0.630 |
|  | 3 - 4a/b | 0.0014 | 0.0022 |  | 0.484 |
| ypN | pN1-3 - pN0 | 0.0021 | 0.0009 |  | 0.234 |
| ypM | pM1 - pM0 | 0.0029 | 0.0014 |  | 0.601 |
| R status | R0 - R1 | 0.0016 | 0.0013 |  | 0.968 |
| Pathological regression | 2/3 - 1a/1b | 0.0016 | 0.0014 |  | 0.594 |

**Supplementary methods**

**Clinical data**

The following conditions were considered severe: Decompensated renal insufficiency, decompensated cardiac insufficiency, liver cirrhosis, status post (s/p) myocardial infarction, s/p valve replacement, s/p stroke, s/p carotid stenosis, severe coronary heart disease, complicated diabetes mellitus, chronic pancreatitis, Chronic Obstructive Pulmonary Disease (COPD), or lung emphysema.

**Immunhistochemical stainings**

The immunohistochemical double stainings were performed on 4 μm FFPE whole slide tissue sections from the primary tumor and the paired normal tissue following standard methodologies. Briefly, after deparaffination and rehydration, antigen retrieval was performed for 20 minutes with 6mMol/l citrate buffer, pH6. Endogenous peroxidase was blocked with 3% hydrogen peroxide in methanol for 10min. Nonspecific binding was blocked with Universal Power Blocking Reagent (10x, BioGenex Laboratories) for 45 minutes. Incubation with first primary antibodies was performed overnight, at 4 °C. Sections were then incubated with the secondary antibody for 45 minutes followed by the DAB detection system. Subsequently the sections were incubated with the second primary antibodies again overnight at 4℃ followed by the incubation with the secondary antibodies for another 45 minutes. Afterwards the sections were stained with Vector red substrate. The following primary antibodies were used for antigen detection: anti-ADRB2 (1:300, Abbiotech Cat# 251604 and anti-PGP9.5 (1:500, Abcam Cat# ab8189). As a positive control human pancreatic tissue was used for anti-ADRB2 and human brain tissue for anti-PGP9.5. A second tissue section was placed on every slide as a negative control and was treated identically, but without the primary antibodies. Every antibody was stained twice per patients and the highest value was taken for further analysis. All slides were counterstained with hematoxylin and reviewed by a senior pathologist with experience in gastrointestinal tumors and the tumor section was marked.

The semiquantative analysis was done via QuPath using a standard protocol^57, 58^ . All slides were digitized using Hamamatsu NanoZoomer S60 Brightfield-Scan at 40x magnification and the scripts per marker are added at the end of this section. Briefly, images were set to brightfield with Hematoxylin and DAB stains. Color deconvolution was applied with specified optical density values for accurate stain separation. The tumor area of the stainings were manually annotated based on the previous annotation from the pathologist. The Positive Cell Detection plugin was used with optical density sum for detection, a pixel size of 0.5 microns, and an 8.0-micron background correction radius. For ADRB2 analysis detection thresholds were set for DAB optical density max in the cytoplasm, with positive staining thresholds at 0.25. For the PGP9.5 analysis a pixel-classifier was manually trained and used to detect the Vector red positive stained cells. Post-processing included watershed and boundary smoothing. The number of positive cells and area detected were used to calculate the average number of positive cells in percent in the tumorbed. For analysis and representation in the tables, the percentages are presented as decimals (e.g., 31% is represented as 0.31).

Skript ADRB2

setImageType('BRIGHTFIELD_H_DAB');

setColorDeconvolutionStains('{"Name" : "H-DAB modified", "Stain 1" : "Hematoxylin", "Values 1" : "0.65111 0.70119 0.29049", "Stain 2" : "DAB", "Values 2" : "0.18399 0.43998 0.87896", "Background" : " 255 255 255"}');

selectAnnotations();

runPlugin('qupath.imagej.detect.cells.PositiveCellDetection', '{"detectionImageBrightfield":"Optical density sum","requestedPixelSizeMicrons":0.5,"backgroundRadiusMicrons":8.0,"backgroundByReconstruction":true,"medianRadiusMicrons":0.0,"sigmaMicrons":1.5,"minAreaMicrons":10.0,"maxAreaMicrons":400.0,"threshold":0.17,"maxBackground":1.0,"watershedPostProcess":true,"excludeDAB":false,"cellExpansionMicrons":5.0,"includeNuclei":true,"smoothBoundaries":true,"makeMeasurements":true,"thresholdCompartment":"Cytoplasm: DAB OD max","thresholdPositive1":0.25,"thresholdPositive2":0.4,"thresholdPositive3":0.6000000000000001,"singleThreshold":false}')

Skript PGP9.5

setImageType('BRIGHTFIELD_H_DAB');

setColorDeconvolutionStains('{"Name" : "H-DAB default", "Stain 1" : "Hematoxylin", "Values 1" : "0.65111 0.70119 0.29049", "Stain 2" : "DAB", "Values 2" : "0.26917 0.56824 0.77759", "Background" : " 255 255 255"}');

selectAnnotations();

runPlugin('qupath.imagej.detect.cells.WatershedCellDetection', '{"detectionImageBrightfield":"Optical density sum","requestedPixelSizeMicrons":0.5,"backgroundRadiusMicrons":8.0,"backgroundByReconstruction":true,"medianRadiusMicrons":0.0,"sigmaMicrons":1.5,"minAreaMicrons":10.0,"maxAreaMicrons":400.0,"threshold":0.17,"maxBackground":1.0,"watershedPostProcess":true,"excludeDAB":false,"cellExpansionMicrons":5.0,"includeNuclei":true,"smoothBoundaries":true,"makeMeasurements":true}')

addPixelClassifierMeasurements("nerve3 (3)", "nerve3 (3)")

classifyDetectionsByCentroid("nerve3 (3)")
