# Supplementary figures and images for: β-Blockers Influence Oncological Outcomes in Gastric Cancer Patients Treated with Neoadjuvant Chemotherapy Based on the Pathological Subtype: A Retrospective Cohort Study
Source: Ann Surg Oncol. 2025 Mar 25;32(7):5142–53. doi: 10.1245/s10434-025-17233-9 (PMC12129865; doi:10.1245/s10434-025-17233-9)

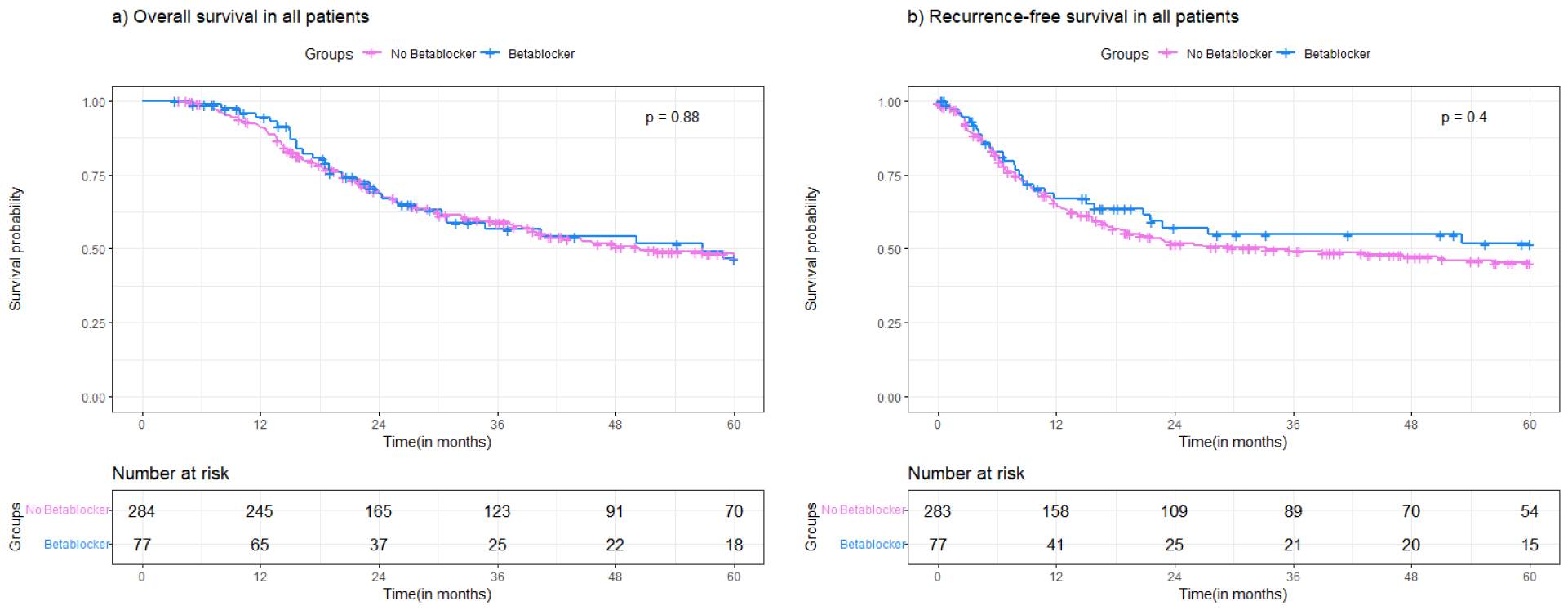

Supplement: Supplementary file 2 — Supplementary file2 (TIF 4690 KB) [file 10434_2025_17233_MOESM2_ESM.tif]
